# Supplementary material for: The Validation of the Persian Version of the ID-Migraine Questionnaire
Source: Med Sci (Basel). 2025 Oct 1;13(4):213. doi: 10.3390/medsci13040213 (PMC12551042; doi:10.3390/medsci13040213)
Supplement: Supplementary file 1 [file medsci-13-00213-s001.zip › medsci-3850498-supplementary.pdf]

Supplementary 1. Items from the original ID Migraine and Persian version of ID Migraine		
	Original ID Migraine	Persian version of ID Migraine
Heading	During the last 3 months did you have the following with your headaches	آیا در طول 3 ماه گذشته، موارد زیر را همراه با سردردهای خود داشته اید؟
Item 1	You felt nauseated or sick to your stomach?	آیا احساس تهوع یا ناخوشی در معده خود داشتید؟
Item 2	Light bothered you (a lot more when you did not have headaches)?	آیا نور شما را آزار می داد؟ (خیلی بیشتر از زمانیکه سردرد نداشتید)
Item 3	Your headaches limited your ability to work, study, or do what you needed to do for at least 1 day?	آیا سردردهایتان، توانایی شما را برای کار، مطالعه یا انجام کارهایی که باید انجام می دادید بمدت حداقل 1 روز محدود می کرد؟
